# Evolutionary engineering strategies to enhance tolerance of xylose utilizing recombinant yeast to inhibitors derived from spruce biomass

**DOI:** 10.1186/1754-6834-5-32

**Published:** 2012-05-11

**Authors:** Rakesh Koppram, Eva Albers, Lisbeth Olsson

**Affiliations:** 1 Department of Chemical and Biological Engineering, Industrial Biotechnology, Chalmers University of Technology, Göteborg, SE-412 96, Sweden; 2 Taurus Energy AB, Ideon, Ole Römers väg 12, Lund, SE-223 70, Sweden

**Keywords:** Lignocellulose, Inhibitors, Evolutionary engineering

## Abstract

**Background:**

One of the crucial factors for a sustainable and economical production of lignocellulosic based bioethanol is the availability of a robust fermenting microorganism with high tolerance to inhibitors generated during the pretreatment of lignocellulosic raw materials, since these inhibitors are known to severely hinder growth and fermentation.

**Results:**

A long-term adaptation in repetitive batch cultures in shake flasks using a cocktail of 12 different inhibitors and a long-term chemostat adaptation using spruce hydrolysate were used as evolutionary engineering strategies to improve the inhibitor tolerance in the metabolically engineered xylose utilizing *Saccharomyces cerevisiae* strain, TMB3400. The yeast was evolved for a period of 429 and 97 generations in repetitive batch cultures and chemostat cultivation, respectively. During the evolutionary engineering in repetitive batch cultures the maximum specific growth rate increased from 0.18 h^-1^ to 0.33 h^-1^ and the time of lag phase was decreased from 48 h to 24 h. In the chemostat adaptation, after 97 generations, the specific conversion rates of HMF and furfural were found to be 3.5 and 4 folds higher respectively, compared to rates after three generations. Two evolved strains (RK60-5, RKU90-3) and one evolved strain (KE1-17) were isolated from evolutionary engineering in repetitive batches and chemostat cultivation, respectively. The strains displayed significantly improved growth performance over TMB3400 when cultivated in spruce hydrolysate under anaerobic conditions, the evolved strains exhibited 25 to 38% increase in specific consumption rate of sugars and 32 to 50% increased specific ethanol productivity compared to TMB3400. The evolved strains RK60-5 and RKU90-3 were unable to consume xylose under anaerobic conditions, whereas, KE1-17 was found to consume xylose at similar rates as TMB3400.

**Conclusion:**

Using evolutionary engineering strategies in batch and chemostat cultivations we have generated three evolved strains that show significantly better tolerance to inhibitors in spruce hydrolysate and displayed a shorter time for overall fermentation of sugars compared to the parental strain.

## Background

Increasing the use of biofuels such as bioethanol for energy generation is of great interest nowadays because they allow alleviation of green house gas production and supply means of energy security in longer perspective [1]. The raw material for bioethanol production should come from non-edible biomass to avoid competition between feedstocks for food and fuel production. One renewable and abundant energy resource that can possibly displace a significant portion of petroleum usage is ethanol produced from lignocellulosic biomass derived from forest and agricultural by-products [1-3]. Forestry biomass such as spruce, birch and biomass from agricultural by-products such as wheat straw and sugarcane bagasse are some of the potential candidates for lignocellulosic ethanol production [4]. Softwoods, predominantly spruce represent one of the major forest reserves in the Nordic countries. It has high carbohydrate content and using such carbohydrate-rich side streams for ethanol production would contribute to the diversification and intensification of the forest industry [5]. The polysaccharides present in lignocellulosic materials are embedded in a recalcitrant and inaccessible structure that needs to be broken up to convert them to fermentable sugars. One of the commonly used pretreatment methods to disrupt the structure of lignocellulosic materials is steam explosion, with the addition of H_2_SO_4_ or SO_2_, which removes most of hemicellulose, followed by enzymatic hydrolysis to convert cellulose to glucose [6]. During pretreatment the release of sugar monomers is often accompanied by liberation of compounds that inhibits growth and metabolism of fermenting microorganism. Some of the inhibitory compounds are present in the wood and others are formed from degradation of sugar and lignin molecules during pretreatment [7]. These inhibitory products are broadly classified into three categories: furans, weak acids and phenolics as reviewed by Palmqvist and Hahn-Hägerdal [8]. Extensive research have been performed to study the inhibition effects on fermenting microorganisms, an elaborate review on formation of inhibitors, their mechanistic action and effect on *S. cerevisiae* has been presented by Almeida *et al*. [9]. Furans such as 2-furaldehyde (furfural) and 5-hydroxymethyl-2-furaldehyde (HMF) inhibit the oxidative metabolism and fermentation of yeasts [10]. Presence of weak acids such as acetic, formic and levulinic acid have shown to result in significantly decreased ethanol yield and productivity in *S. cerevisiae* fermentations [11,12]. Phenolics include a wide variety of aromatic alcohols, aldehydes and acids, some of them notably include catechol, coniferyl alcohol, coniferyl aldehyde, vanillin, syringaldehyde, hydroquinone, cinnamic acid, p-coumaric acid and these compounds have been shown to limit the growth of *S. cerevisiae* and ethanol formation [13], however, the mode of action of these weak acids and phenolics on microbial physiology still remains unclear due to their molecular heterogeneity and lack of qualitative and quantitative analyses of high accuracy. Several detoxification methods such as alkali treatment, sulfite treatment, evaporation, anion exchange and treatment with laccase have been used to remove or decrease the level of inhibitory compounds in lignocellulosic hydrolysate leading to improved fermentability, however, these methods also resulted in loss of fermentable sugars [14]. Furthermore, it has been shown that detoxification cost constitutes as much as 22% of total ethanol production cost which economically limits their use [15]. One of the possible alternatives to circumvent inhibitor problems is to improve the fermenting microorganism by long term adaptation to inhibitors present in lignocellulosic hydrolysates. In laboratory conditions, evolutionary engineering is usually a phenomenon of long term adaptation of cells under selective pressure, where variants of cell population with a selective advantage exponentially take over the initially dominating cells [16]. The advantage of using evolutionary engineering is that a detailed understanding of the action of inhibitors and their complex nature of interaction with biochemical networks is not needed [9]. Some notable approaches include evolution of a fermenting microorganism to a whole hydrolysate containing high content of inhibitors [17] or to a defined media supplemented with one or more synthetic inhibitors [18]. The advantage of using synthetic inhibitors is better regulation of individual inhibitor concentrations and the ability to study their effect on cell growth, ethanol yield and productivity. It is evident from earlier studies that the combination of different inhibitors has more pronounced effect on cells due to their synergistic behavior [19]. However, evolution to a cocktail of inhibitors from three different categories has to the best of our knowledge not been investigated before and will be one of the focus of our work describe here. *S. cerevisiae* is a well characterized microorganism that has traditionally been used for ethanol production from hexoses providing high yields and productivities, in addition to high ethanol tolerance. Furthermore, it has an innate ability to metabolize HMF and furfural to the less inhibitory compounds 5-hydroxymethyl furfuryl alcohol and furfuryl alcohol, respectively [10,20,21]. However, on the downside, *S. cerevisiae* cannot utilize xylose, an abundant pentose sugar in lignocellulosic hydrolysates [22]. Several metabolic engineering strategies have been performed for the development of recombinant *S. cerevisiae* strains to utilize and ferment xylose [23-25]. Previous investigations have demonstrated that a combination of metabolic engineering and mutagenesis was successful in developing superior recombinant xylose utilizing *S. cerevisiae*[26]. In the present study, we used a xylose utilizing recombinant *S. cerevisiae*[26] as the initial strain to enhance inhibitor tolerance in two modes of evolutionary engineering, one in repetitive batch cultures of defined medium containing a cocktail of inhibitors that are constituent of spruce hydrolysate and another in chemostat cultures using spruce hydrolysate. The aims of the present work were to compare the fermentative performance of evolved strains with that of parental strain using spruce hydrolysate and to compare the outcome of two modes of evolutionary engineering.

## Results

### Evolutionary engineering in repetitive batch cultures with inhibitor cocktail

**Figure 1:**
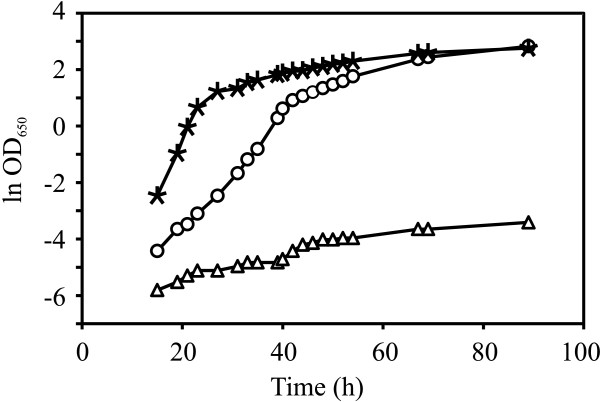
**Inhibitory effects of inhibitor cocktail on*S. cerevisiae*TMB3400.** Growth curves of *S. cerevisiae* TMB3400 in the presence of different concentrations of inhibitor cocktail in minimal medium containing 20 g l^-1^ of glucose and xylose, respectively. No inhibitors (asterisks), 20% inhibitor cocktail (circles), 100% inhibitor cocktail (triangles).

A cocktail of 12 different inhibitors spanning three different inhibitor categories: furans, weak acids and phenolics (Table 1) were used to evolve *S. cerevisiae* TMB3400 in repetitive batch cultures aiming at enhancing its inhibitor tolerance. Initially, the innate level of inhibitor tolerance of the strain TMB3400 was tested by cultivating in minimal medium containing various concentrations of inhibitor cocktail. The growth was followed by measuring the optical density at different time intervals. In presence of 100% inhibitor cocktail the μ_max_ was estimated to 0.03 h^-1^, however, in presence of 20% inhibitor cocktail the strain was able to grow at a μ_max_ of 0.18 h^-1^ which was 55% lower compared to μ_max_ of 0.40 h^-1^ in absence of inhibitors (Figure 1). Considering the scope for improvement, the evolution was initiated in presence of 20% inhibitor cocktail and gradually the concentration was increased in steps of 20%. The progressive development during the evolutionary engineering was followed as a function of increased μ_max_ and reduced lag phase time.

**Table 1:** Composition of pretreated spruce hydrolysate and inhibitor cocktail solution

**Compound**	**Spruce hydrolysate, g l**^**-1**^	**Inhibitor cocktail, g l**^**-1**^
Glucose	18	-
Mannose	16	-
Galactose	4.5	-
Xylose	9	-
Arabinose	4	-
HMF	3.4	3.4
Furfural	1.1	1.1
Acetic acid	6.3	6.3
Formic acid	1.2	1.2
Levulinic acid	2.4	2.4
Vanillin	0.11	0.14
Syringaldehyde	<0.001	0.12
Coniferyl aldehyde	NA	0.05
Hydroquinone	NA	0.02
Cinnamic acid	NA	0.001
4-Hydroxybenzoic acid	NA	0.02
Guaiacyl acetone	NA	0.02

NA: not analyzed.

#### 20% inhibitor cocktail

**Figure 2:**
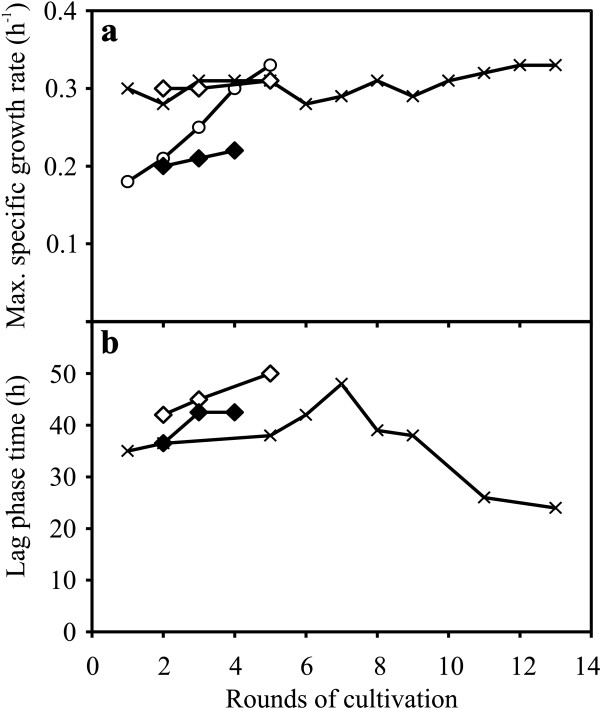
**Evolutionary progress in repetitive batch culture.** Progression of (**a**) maximum specific growth rate and (**b**) lag phase time profile during evolutionary engineering in repetitive batch cultures at different levels of inhibitor cocktail in minimal medium containing 20 g l^-1^ of glucose and xylose, respectively. 20% inhibitor cocktail (circles), 40% inhibitor cocktail (crosses), 60% inhibitor cocktail (diamonds), UV treated cells in 60% inhibitor cocktail (closed diamonds).

Under a selective pressure of 20% inhibitor cocktail five rounds of repetitive batch cultures were performed, in which cells were evolved for 48 generations. The μ_max_ was found to be progressively increased during the course of evolution and at the end of the five rounds μ_max_ was calculated to be 0.33 h^-1^, which was a substantial increase by 89% compared to the initial μ_max_ of 0.18 h^-1^ (Figure 2a). There was hardly any lag phase observed during the evolution in presence of 20% inhibitor cocktail.

#### 40% inhibitor cocktail

**Figure 3:**
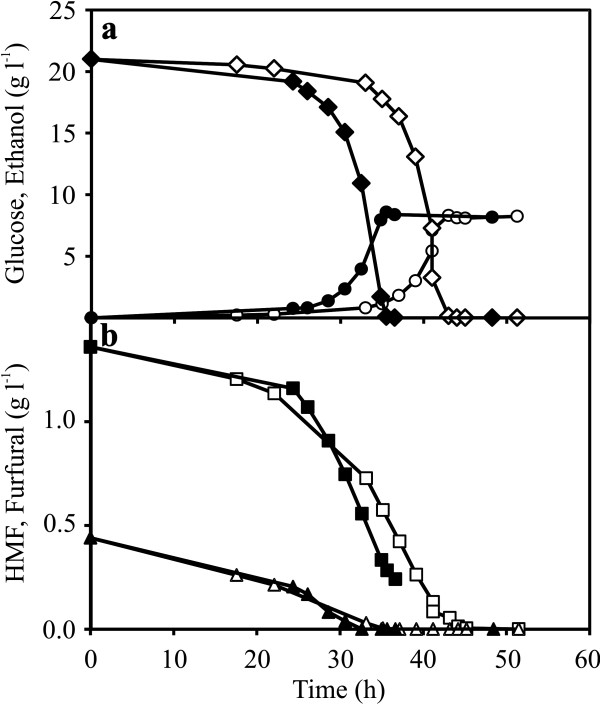
**Progression of glucose consumption, ethanol production and inhibitors conversion, in repetitive batch culture.** (**a**) glucose consumption and ethanol production, (**b**) Conversion of HMF and furfural by RK40-3 (open symbols) and RK40-13 (closed symbols) during the evolution in 40% inhibitor cocktail in minimal medium containing 20 g l^-1^ of glucose and xylose, respectively. Glucose (diamonds), ethanol (circles), HMF (squares), furfural (triangles).

The evolution was directed in presence of 40% inhibitor cocktail with the strain (RK20-5) evolved for five rounds in presence of 20% inhibitor cocktail. Thirteen rounds of repetitive batch cultures were performed for a total of 265 generations. There was no observed significant improvement in μ_max_ that remained nearly constant at 0.30 h^-1^ for every round of cultivation (Figure 2a). However, an interesting lag phase profile was observed. Initially, the lag phase time increased for every round and reached a peak in the seventh round of cultivation, later, gradually reduced to 24 h in the thirteenth round of cultivation, which was an improvement of 50% compared to the peak value of 48 h (Figure 2b). This was well justified from the analysis of extracellular samples collected during the evolution process. For instance, cells from third round of cultivation (RK40-3) and thirteenth round of cultivation (RK40-13) were compared for HMF and furfural conversion, glucose consumption and ethanol production (Figure 3). There was a significant improvement in fermentation time in terms of glucose consumption and ethanol production. RK40-13 consumed all glucose and reached maximum ethanol concentration (8.6 g l^-1^) in 35 h, whereas, RK40-3 consumed all glucose and reached maximum ethanol concentration (8.3 g l^-1^) in 45 h (Figure 3a). The rate of HMF conversion was increased marginally, however, no significant difference was observed in the rate of furfural conversion (Figure 3b). Also, RK40-3 and RK40-13 consumed 16% and 10% of the supplied xylose, respectively during the time span of 12 h after the depletion of glucose until the subsequent cell transfer (data not shown). To increase genetic variability, cells from twelfth round of cultivation (RK40-12) were subjected to UV irradiation for 30, 60 and 90 seconds, respectively. The growth curve in presence of 40% inhibitor cocktail of strains resulting from 30 and 60 seconds UV exposure were indistinguishable, whereas, strain that was exposed to UV for 90 seconds consumed glucose relatively faster (data not shown).

#### 60% inhibitor cocktail

Directed evolution in presence of 60% inhibitor cocktail was started with RK40-12 exposed to UV for 90 seconds and RK40-13. Two culture lineages were developed, in which UV and non-UV treated cells were evolved in 60% inhibitor cocktail for 84 and 116 generations, respectively. The μ_max_ of the non-UV treated lineage remained nearly constant at 0.30 h^-1^ and the lag phase time increased for every round of cultivation (Figure 2a,b). The UV treated lineage grew slowly with nearly a constant μ_max_ of 0.20 h^-1^ for every round of cultivation; however, with a shorter lag phase time compared to the non-UV treated lineage (Figure 2a,b).

### Evolutionary engineering in continuous culture with spruce hydrolysate

**Figure 4:**
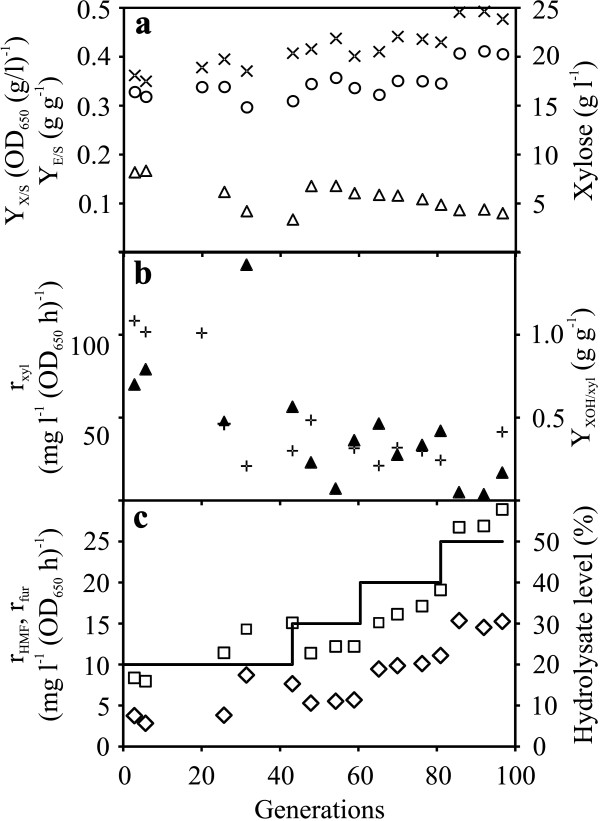
**Evolutionary progress in chemostat culture supplemented with spruce hydrolysate.** (**a**) Yields of biomass (Y _X/S_) and ethanol (Y _E/S_) on consumed sugars (left ordinate) and xylose concentration in the chemostat (right ordinate). Biomass (crosses), ethanol (circles), xylose (triangles). (**b**) Specific consumption rate of xylose (r _xyl_) (left ordinate) and xylitol yield (Y _XOH/xyl_) from consumed xylose (right ordinate). Xylose (closed triangles), xylitol (plus). (**c**) Specific conversion rates of HMF (r _HMF_) and furfural (r _fur_) (left ordinate), line with step wise increase shows the generation points at which the spruce hydrolysate concentration was increased (right ordinate). HMF (squares), furfural (diamonds).

Prolonged chemostat cultivation of the strain TMB3400 in presence of spruce hydrolysate supplemented to minimal medium was performed to improve the conversion rate of the growth- affecting inhibitors in order to enhance the inhibitor tolerance. As a measure of growth, the optical density was followed during the chemostat, when OD_650_ increased the selection pressure for adaptation was also increased by increasing the concentrations of spruce hydrolysate in steps of 10% (v/v). Initially the evolution was started with 20% spruce hydrolysate with a dilution rate of 0.08 h^-1^, which was reduced to 0.05 h^-1^ for hydrolysate concentrations higher than 20%. The cells were evolved for 97 generations (60 days) in presence of increasing concentrations of spruce hydrolysate before the chemostat was terminated. Throughout the chemostat, the residual glucose concentration remained similar at a low level below 0.5 g l^-1^, only 5 to 10% of the supplied xylose was consumed during the first 40 generations, and later, xylose gradually accumulated (Figure 4a). The reduced xylose consumption capacity was also seen from 8 fold decrease in the specific xylose consumption rate during the progress of the evolution (Figure 4b). A significant improvement in ethanol yield from 0.32 to 0.41 g g^-1^ on consumed sugars was observed during the evolution and as a consequence, there was a gradual decrease in biomass yield on consumed sugars (Figure 4a). From the measured HMF and furfural concentrations it was observed that about 80 to 85% of the supplied HMF and furfural were converted during the cultivation at 20% or 30% levels of spruce hydrolysate (data not shown). However, at higher levels the conversion was reduced, 65% and 20% of the supplied HMF was converted during the cultivation at 40% and 50% levels of spruce hydrolysate, respectively. Similarly, 70% and 50% of the supplied furfural was converted during the cultivation at 40% and 50% levels of hydrolysate, respectively. The fact that less HMF could be converted by the cells at higher levels of hydrolysate was also seen from the concentration ratio of HMF to furfural, which increased from 2.8 at the 20-30% level to 3.7 and 4.7 at 40% and 50% of hydrolysate, respectively. At generation 31, the specific conversion rates of HMF and furfural was two and three fold higher than at generation 6, respectively, and this trend of increasing specific conversion rates of furans was found throughout the adaptation, indicating the evolutionary progress toward improved HMF and furfural conversion (Figure 4c). Acetate present in the hydrolysate was consumed to some extent by the cells but at the end of the evolutionary regime with 50% hydrolysate the specific consumption rate of acetate was decreased by 35% compared to the consumption rate at the beginning of the cultivation (data not shown). This also support that at higher hydrolysate levels the inhibitors started affecting the cellular performance.

### Screening and selection of evolved strains for characterization

Evolved cells sampled at different time points during the process of evolutionary engineering were screened by drop tests. Six ten-fold dilutions of cell suspension were incubated on YP-hydrolysate and YPD75 plates. All evolved cells from repetitive batch cultures performed better than parental strain TMB3400. Notably, cell population of two lineages (UV and non-UV treated) evolved in 60% inhibitor cocktail had relatively higher number of colonies across the dilution series on YP- hydrolysate plate than TMB3400 (data not shown). YPD75 plate containing 75% inhibitor cocktail was found to be more inhibitory than YP-hydrolysate, in particularly to TMB3400 with very few colonies on the first dilution and no colonies appearing on rest of the dilutions. Whereas, cells evolved in 60% inhibitor cocktail showed relatively more colonies, however, only on the first two dilutions. Selection of cell population for further characterization was primarily based on their ability to grow on inhibitor plates such as YPD75 and YP-hydrolysate. Since the performance of cells evolved in 60% inhibitor cocktail was better than TMB3400, two strains, RK60-5 (evolved in 60% inhibitor cocktail for 5 rounds of cultivation) and RKU90-3 (UV exposure for 90 seconds and evolved in 60% inhibitor cocktail for 3 rounds of cultivation) were chosen for further physiological characterization. Evolved cells from chemostat cultivation were compared with TMB3400 and there was hardly any notable difference observed in number of colonies on YP-hydrolysate plate. Accordingly, we chose to consider other factors for selection of cell populations. The cell population after 97 generations exhibited the highest ethanol yield of 0.41 g g^-1^ consumed sugars and was evolved in chemostat for longest duration (Figure 4a). The cell population at generation 97 was designated as KE1-17. During the chemostat cultivation, xylose gradually accumulated after 40 generations (Figure 4a), therefore, we compared xylose consumption and xylitol produced from consumed xylose at various generation points during the evolution. At generation 31, the specific xylose consumption rate was at the highest at 142 mg l^-1^ OD_650-1_ h^-1^ and the xylitol yield was at the lowest at 0.21 g g^-1^ consumed xylose (Figure 4b). This cell culture at generation 31 was designated as KE1-5. KE1-17 and KE1-5 from the chemostat adaptation were chosen for further physiological characterization.

### Fermentation characteristics of evolved strains

The fermentation performances of the selected strains were compared in anaerobic batch cultures in minimal medium and spruce hydrolysate. Anaerobic fermentation in minimal medium was performed with 20 g l^-1^ glucose and xylose each as carbon source. To quantitatively compare the fermentation performance, physiological parameters were calculated and it was found that all the evolved strains, but KE1-5, performed better than parental strain TMB3400. The strains RK60-5, RKU90-3 and KE1-17 exhibited 10 to 20% higher μ_max_ than TMB3400, whereas, KE1-5 displayed 8% lower μ_max_ than TMB3400 (Table 2). All the strains displayed maximum ethanol concentrations ranging between 8.2 to 8.7 g l^-1^ and the ethanol yields for all evolved strains showed a marginal increase of 3 to 5% higher than TMB3400. However, the specific glucose consumption rate and specific ethanol productivity of RK60-5, RKU90-3 and KE1-17 were significantly increased by 16 to 32% compared to TMB3400 (Table 2). It was observed for TMB3400, KE1-5 and KE1-17 strains that during the initial exponential growth on glucose, almost no xylose was consumed, as soon as glucose was depleted, growth ceased and cells started to utilize xylose. The specific xylose consumption rate calculated after the depletion of glucose, was significantly reduced by 50% in KE1-5 (0.05 g g cell dry weight^-1^ h^-1^) and 20% in KE1- 17 (0.08 g g cell dry weight^-1^ h^-1^) compared to the value for TMB3400 (0.10 g g cell dry weight^-1^ h^-^^1^). Also, the xylitol yields for KE1-5 (0.74 g g consumed xylose^-1^) and KE1-17 (0.66 g g consumed xylose^-1^) were higher than the yield exhibited by TMB3400 (0.61 g g consumed xylose^-1^). Surprisingly, in anaerobic batch cultures of RK60-5 and RKU90-3, when the glucose was depleted, xylose was hardly utilized even after 100 h of fermentation and minute traces of xylitol (0.3 g l^-1^) were observed.

**Table 2:** Physiological characterization: Anaerobic fermentation in minimal medium with 20 g l-1 glucose and xylose, respectively

**Strain**	**μ**_**max**_	**Y**_**X/S**_	**Y**_**E/S**_	**Y**_**gly/S**_	**Y**_**Ac/S**_	**r**_**glu**_	**r**_**xyl**_	**r**_**E**_
TMB3400	0.37±0.01	0.11±0.00	0.35±0.00	0.10±0.00	0.005±0.001	3.35±0.05	0.10±0.00	1.17±0.02
RK60-5	0.42±0.01	0.11±0.00	0.36±0.00	0.10±0.00	0.005±0.000	3.87±0.07	0.00	1.39±0.02
RKU90-3	0.44±0.00	0.11±0.00	0.37±0.00	0.11±0.01	0.005±0.001	4.15±0.02	0.00	1.54±0.02
KE1-5	0.34±0.01	0.11±0.00	0.36±0.01	0.10±0.01	0.007±0.001	3.03±0.05	0.05±0.00	1.08±0.01
KE1-17	0.41±0.01	0.12±0.01	0.36±0.01	0.09±0.00	0.007±0.000	3.56±0.06	0.08±0.02	1.26±0.01

The maximum specific growth rate (μ_max_) is represented in h^-1^. The specific consumption rate of glucose (r _glu_), xylose (r _xyl_) and specific ethanol productivity (r _E_) are represented in g g cell dry weight^-1^ h^-1^. The yield of biomass (Y _X/S_), ethanol (Y _E/S_), glycerol (Y _gly/S_), and acetate (Y _Ac/S_) are represented in g g consumed sugars^-1^. All values are calculated during the exponential phase identified by natural logarithm of biomass and culture time. The values are average from duplicate experiments and ± indicates deviation from the mean.

To further elucidate the phenotypic differences between evolved strains and TMB3400, anaerobic fermentations were performed in spruce hydrolysate containing carbon sources such as glucose, mannose, galactose, xylose and arabinose, the composition of which is presented in the Table 1. It was observed in all fermentations that the sugars glucose and mannose were co-utilized and when glucose was depleted, galactose was consumed. Xylose consumption varied between the strains, TMB3400 utilized 15 to 17% of supplied xylose, KE1-5 consumed 10 to 12% and KE1-17 metabolized only 5% during 70 h of fermentation. There was hardly any xylose consumed by the RK60-5 and RKU90-3 strains. Arabinose was untouched by all the strains. The physiological parameters were determined and it was found that RK60-5 and RKU90-3 displayed the highest μ_max_ of 0.10 h^-1^ which was 43% higher than TMB3400 (Table 3). The ethanol yields displayed by the evolved strains were 8 to 10% higher than TMB3400. The strains RK60-5 and RKU90-3 exhibited a significant increase of 38% and 50% in specific sugar consumption rate and specific ethanol productivity, respectively. Similar improvement in specific ethanol productivity was observed in KE1-17, while sugar consumption and ethanol production rate of KE1-5 were similar to that of TMB3400. Surprisingly, there were no significant differences observed in rates of inhibitor conversion. The specific conversion rates of HMF and furfural were similar among all the strains with the exception being KE1-17 which showed 57% increase in the specific conversion rate of furfural compared to the strain TMB3400 (Table 3).

**Table 3:** Physiological characterization: Anaerobic fermentation in spruce hydrolysate

**Strain**	**μ**_**max**_	**Y**_**X/S**_	**Y**_**E/S**_	**Y**_**gly/S**_	**r**_sugars*_	**r**_**E**_	**r**_**HMF**_	**r**_**fur**_
TMB3400	0.07±0.01	0.07±0.01	0.40±0.02	0.05±0.01	1.09±0.17	0.44±0.07	0.08±0.00	0.07±0.01
RK60-5	0.10±0.01	0.07±0.00	0.44±0.00	0.04±0.00	1.50±0.01	0.66±0.01	0.08±0.01	0.08±0.01
RKU90-3	0.10±0.01	0.07±0.01	0.43±0.01	0.04±0.01	1.49±0.01	0.63±0.01	0.07±0.00	0.08±0.00
KE1-5	0.07±0.00	0.07±0.01	0.44±0.02	0.03±0.01	1.07±0.20	0.47±0.11	0.08±0.01	0.07±0.01
KE1-17	0.08±0.00	0.06±0.00	0.43±0.00	0.02±0.00	1.36±0.09	0.58±0.03	0.09±0.01	0.11±0.00

The maximum specific growth rate (μ_max_) is represented in h^-1^. The specific consumption rate of sugars (r _sugars_), specific conversion rate of HMF (r _HMF_), furfural (r _fur_) and specific ethanol productivity (r _E_) are represented in g g cell dry weight^-1^ h^-1^. The yield of biomass (Y _X/S_), ethanol (Y _E/S_) and glycerol (Y _gly/S_) are represented in g g consumed sugars^-1^. All values are calculated during the exponential phase identified by natural logarithm of biomass and culture time. The values are average from duplicate experiments and ± indicates deviation from the mean. ^*^Specific consumption rate of glucose and mannose, since during the exponential phase glucose and mannose are co-consumed.

### Enzyme activities

The evolved strains RK60-5 and RKU90-3 were unable to consume xylose during anaerobic fermentations in presence of glucose and xylose. Specific activities of enzymes XR and XDH of RK60-5 and RKU90-3 were compared with TMB3400 grown in presence of 20 g l^-1^ glucose and xylose, respectively. The recombinant *S. cerevisiae* strain TMB3400 expressed XR and XDH with activities of 0.20±0.00 and 1.23±0.34 U mg protein^-1^. Interestingly, there were no activities of XR and XDH detected in cell extracts of evolved strains RK60-5 and RKU90-3.

## Discussion

### Influence of evolutionary strategies on selection parameters

The lag phase time, μ_max_ and inhibitor consumption rates were chosen as selection parameters for inhibitor tolerance. An important characteristic of evolution in repetitive batch culture is cells go through distinct phases of lag, exponential, transition and stationary growth under a given selection pressure. Evolution in chemostat culture provides a constant environment for the cells to adapt to. Evolutionary events that arise from advantages in any of these phases in batch and chemostat cultures may influence the fitness of cells [27]. The two evolutionary engineering strategies employed here were found to affect the selection parameters differently. Evolution in repetitive batch cultures influenced the maximum specific growth rate, specific glucose consumption rate and ethanol productivity whereas, evolution in chemostat cultures influenced specific conversion rates of inhibitors. For the first time, evolutionary engineering to enhance tolerance towards the synergistic action of inhibitors using a cocktail of 12 different inhibitors was demonstrated for a recombinant xylose- utilizing strain of *S. cerevisiae*. The lag phase, commonly referred as a preparatory phase for the cell growth was not affected in presence of 20% inhibitor cocktail. However, in presence of 40% inhibitor cocktail, a prolonged lag phase and a feature of increasing lag phase time followed by decreasing lag phase time was observed during the time course of the evolution process (Figure 2b).

The prolonged lag phase before the recovery of cell growth indicates a major shift in physiology of cells evolved under inhibitor stress. This interesting lag phase profile can be speculated to be due to accumulation of cell populations with non-beneficial mutations until they are overgrown by the cell population with beneficial mutations. Evolution in repetitive batch cultures clearly favored the improvement of μ_max_ (Figure 2a and Table 3). The specific conversion rates of HMF and furfural were less affected during the evolution in batch cultures, however in contrast, evolution in chemostat cultures clearly favored the improvement of specific conversion rates of HMF and furfural from generation 50 until generation 97 (Figure 4c). Previous evolutionary engineering study in batch and continuous culture for acetic acid tolerance in xylose fermenting *S. cerevisiae* strain reported that acetic acid tolerance was acetate inducible, so when cells were precultured in absence of acetic acid stress they failed to grow in anaerobic batch culture supplemented with acetic acid [28]. However, the evolved strains selected in this study displayed improved performance in anaerobic batch fermentation even though the cells were precultured in minimal medium in absence of inhibitors which suggests that the inhibitor tolerance developed was not inhibitors inducible. The results of fermentation performance in spruce hydrolysate indicated that the strains RK60-5, RKU90-3 displayed marginally higher μ_max_ and all the evolved strains exhibited higher specific ethanol productivity compared to TMB3400 (Table 3). It has been previously shown that growth and ethanol productivity of inhibitor tolerant strain was improved due to higher capacity for reduction of HMF and furfural compared to a less tolerant strain [29]. However, in the anaerobic fermentation of spruce hydrolysate, there was no evident difference observed in the specific conversion rates of HMF and furfural among all the strains except KE1-17. The indistinguishable conversion rate of furfural by evolved and parental strains has been previously reported and mentioned that improved performance of evolved strain was due to increased viability under furfural stress [30]. In screening and selection after evolutionary engineering experiments, our evolved strains grew well and displayed higher number of colonies across the dilution series compared to the strain TMB3400 when incubated on inhibitor plates which suggests increased viability of our evolved strains. Another possible explanation of similar specific conversion rates of HMF or furfural among evolved strains may presumably be explained that the rate of transport of HMF and furfural in to the cells was not affected by evolutionary engineering; however, we speculate that increase in μ_max_ and specific ethanol productivity of evolved strains in spruce hydrolysate could be due to higher in vivo rates of conversion of HMF, furfural and possibly also other inhibitory aldehydes which ensures that the intracellular concentrations of these inhibitors remain low.

### Inhibitor tolerance Vs xylose consumption

The progress in μ_max_ and lag phase time until the evolution in 60% inhibitor cocktail, by no means represents the final stage of evolution. However, as the evolution progressed, the xylose utilization during the time span of 12 h after glucose depletion until cell transfer was reduced significantly, and therefore the evolution in repetitive batches was terminated. Decreased specific xylose consumption rates were also observed during the evolution in chemostat (Figure 4b) therefore, the chemostat was also terminated when the cells were evolving in 50% levels of spruce hydrolysate. The cofactor NADPH dependent HMF and furfural reduction has been previously observed in *S. cerevisiae*[31,32]. The engineered *S. cerevisiae* strain TMB3400 expressing the XR/XDH pathway has been known to require NADPH for xylose reductase activity [23]. Long term exposure of these aldehyde inhibitors evolutionarily favors the yeast cells to use NADPH for furan reduction over xylose reduction since xylose was not the limiting carbon source in our experimental system. The genes XYL1 and XYL2 encoding XR and XDH, respectively, are under the control of constitutive promoters, aldehyde dehydrogenase (ADH) and PGK promoters, respectively. So, the burden for the cells to keep these genes expressed is higher if cultivated in presence of inhibitors for a prolonged time, since the NADPH is directed to inhibitors reduction over xylose reduction. This is well in accordance that there were no activities of XR and XDH enzymes detected from the cell extracts of RK60-5 and RKU90-3 strains. One possible explanation for this observed phenotype of RK60-5 and RKU90-3 is that the XYL1 and XYL2 genes cassette of xylose utilization pathway may have been lost during the process of evolutionary engineering. Although there was a marked reduction in specific xylose consumption rate of strains KE1-5, KE1-17 (Table 2) evolved in chemostat cultures, the xylose consumption ability was still retained. Previous study on inhibitors adaptation of xylose fermenting *S. cerevisiae* strain that retained xylose consumption ability even after 353 h of adaptation in carbon limited chemostat cultures [17] elucidates the importance of evolution in well controlled chemostat cultures to both retain xylose consumption and further improve inhibitor tolerance. Alternatively, the inability of RK60-5 and RKU90-3 to consume xylose (Table 2) clearly indicates that in order to keep the xylose consumption intact, evolutionary engineering for inhibitor tolerance should be performed with xylose as the limiting carbon source in repetitive batch cultures.

## Conclusion

The potential of evolutionary engineering is more vivid in improving a complex phenotypic trait that requires multi-gene modifications such as inhibitor tolerance. In this study, we have developed evolved *S. cerevisiae* strains that showed improved tolerance to inhibitors in spruce hydrolysate. Although three of the evolved strains performed relatively better, the strains evolved in the inhibitor cocktail stands alone with 50% increase in specific ethanol productivity compared to the performance by the parental strain of S.cerevisiae, TMB3400. From an economical point of view, this is an interesting feature since it reduces overall time required for fermentation. Phenotypic characteristics during the process of evolutionary engineering evidently demonstrated the importance of evolution in repetitive batch cultures and chemostat cultures to improve the maximum specific growth rate and inhibitor conversion rates, respectively. Directed evolution in presence of a comprehensive solution of inhibitor cocktail extends the tolerance limits of yeast cells towards three different inhibitor categories which were apparent from improved performance of evolved strains, even though all the strains had similar HMF conversion rates. In addition, the decrease in specific xylose consumption rates of evolved strains has highlighted the consequence of the choice of medium for evolutionary engineering. Since the type of inhibitors and their concentrations may vary according to the type of lignocellulosic raw materials and pretreatment methods used, our study elucidates the significance of dedicated evolutionary engineering experiments to a specific type of raw materials to develop tailor made strains that can show marked improvements.

## Methods

### Strains

The recombinant xylose utilizing *S. cerevisiae* TMB3400 [26] was used as a parental strain in the evolutionary engineering strategies. The parental strain and strains developed during the course of evolution were stored at −80°C in culture aliquots containing 20% sterile glycerol. Volumes of 100 μl from the vials were used to inoculate precultures.

### Spruce hydrolysate

Spruce hydrolysate was provided by SEKAB E-Technology AB (Örnsköldsvik, Sweden) and prepared from spruce wood chips pre-treated with dilute sulfurous acid (SO_2_ in water) and filtered to remove the solids. The composition of the hydrolysate is presented in the Table 1.

### Inhibitor cocktail

The inhibitor cocktail solution used in this study contained 12 different inhibitors (Table 1). The selection of inhibitors and their concentrations for the preparation of inhibitor cocktail solution was based on the analysis of inhibitors composition in spruce hydrolysate used in this study (Table 1). Also, previously published articles that contained data on inhibitors composition in pretreated spruce [4,7,9,11,14,33-36], formed a basis for our choice of inhibitors and their concentrations for the cocktail solution. For practical ease, a ten times concentrated inhibitor cocktail was prepared by dissolving syringaldehyde, coniferyl aldehyde and vanillin in 5 ml 0.1 M NaOH and was added to milliQ water and the pH was set to 6.5. The rest of the components but HMF were dissolved one by one maintaining the pH at 6.5 throughout the preparation. The inhibitor cocktail was then filter sterilized and stored at −20°C in small aliquots. HMF was directly added to the medium during the evolution process.

### Media

Evolution in shake flasks were carried out in minimal medium containing 20 g l^-1^ of glucose and xylose, respectively and enriched with salts, two folds of trace elements and vitamins according to Verduyn *et al*. [37]. The pH of the medium was adjusted to 6.5 with 1 M NaOH for all shake flask cultivations. The inhibitor cocktail was added to minimal media to achieve final concentrations of 20, 40 and 60% of inhibitors concentration presented in Table 1. The cultivation medium for chemostat cultures was based on minimal medium with 20 g l^-1^ xylose and with increasing levels, 20-50% (v/v), of spruce hydrolysate and 0.1% (v/v) of polypropylene glycol P 2’000 from Fluka as antifoam. Glucose was added to the medium giving a final concentration of 20 g l^-1^ taken into account the amount of glucose already present in the hydrolysate. The pH of the medium was adjusted to 5.0 with 1 M NaOH and was supplemented with salts, two folds of trace elements and vitamins according to Verduyn *et al*. [37]. Anaerobic fermentations in minimal medium and spruce hydrolysate were supplemented with antifoam polypropylene glycol P 2’000 from Fluka at 0.1 and 0.25% (v/v), respectively, along with ergosterol and Tween 80. Ergosterol and Tween 80 were dissolved in boiling absolute ethanol and were added to the medium to a final concentration of 0.01 g l^-1^ and 0.42 g l^-1^, respectively. Three different agar plates YPX, YPD75 and YP-hydrolysate were used for screening strains. All the three plates contained 10 g l^-1^ yeast extract, 20 g l^-1^ peptone and 20 g l^-1^ agar. In addition, YPX plate was supplemented with 20 g l^-1^ xylose and YPD75 plate was supplemented with 20 g l^-1^ glucose and 75% of inhibitors concentration (inhibitor cocktail) in Table 1, whereas, in YP- hydrolysate plate yeast extract, peptone and agar were dissolved in spruce hydrolysate.

### UV mutagenesis

The cell samples stored at −80°C were retrieved and diluted with sterile milliQ water to read an OD_650_ of 0.1 and a volume of 100 μl was spread on three YP-hydrolysate plates. The three plates were subjected to high intensity UV radiation at 302nm from a UV transilluminator TMF26V from UVP for 30, 60 and 90 seconds, respectively and incubated at 30°C until colonies were formed. One colony per plate was taken for further evolutionary engineering.

### Cultivation conditions

#### Evolutionary engineering in repetitive batch cultures

Evolutionary engineering in repetitive batch cultures were carried out in 100 ml shake flasks with a working volume of 50 ml. The cultures were inoculated to an initail OD_650_ of 0.01, incubated at 30°C on an orbital shaker at 180 rpm. Samples were frequently withdrawn for measurement of OD_650_ and analysis of extracellular metabolites. The presence of glucose during the cultivation was monitored using glucose test strips (keto-diabur-test 5000 from Roche) and the cells were serially transferred to subsequent shake flasks at 8 to 12 h after the depletion of glucose. Possible contamination of the cultivation was checked by ocular inspection in microscope. Cell samples during the evolution that usually contain mixed cultures were withdrawn periodically and stored in glycerol vials for further evaluation.

#### Evolutionary engineering in continuous cultures

The directed evolution in chemostat culture was performed in a 1 l fermenter (SARA, Belach Bioteknik, Sweden) with 740 ml working volume (measured at the end of the cultivation) determined by having the outlet pumping fixed at a certain height in the vessel. The conditions of the cultivation were controlled at a temperature of 30°C, a pH of 5.0 with 2M NaOH, and a stirrer speed at 350 rpm and with both gas in- and outlets open to the atmosphere. The culture was run for 60 days and samples for measurements of OD_650_ and extracellular metabolites were taken regularly. Possible contamination of the cultivation was checked by ocular inspection in microscope. Cell samples during the evolution that usually contain mixed cultures were withdrawn periodically and stored in glycerol vials for further evaluation.

#### Anaerobic fermentation

Anaerobic fermentations were carried out in 4 l fermenters (Belach Bioteknik, Sweden) with a working volume of 2 l and the anaerobic condition was maintained by flushing N_2_ at flow rate of 0.4 l min^-1^. The pH was maintained at 5.0 by automatic addition of 2 M NaOH and the temperature at 30°C. The stirrer speed was 500 rpm in minimal medium and set to 400 rpm for fermentation of spruce hydrolysate due to excessive foam formation. Precultures for fermentation were inoculated from glycerol vials containing mixed cultures and grown in minimal medium with 20 g l^-1^ of glucose and xylose respectively, in shake flasks until the late exponential phase. The fermentation of minimal medium and spruce hydrolysate was initiated by inoculating to an initial OD_650_ of 0.01 and 0.05, respectively.

### Drop test

Cells in frozen glycerol vials that contained mixed cultures were thawed and diluted with sterile milliQ water to read an OD_650_ of 1.0. Six ten-fold serial dilutions were made and a volume of 10 μl of each dilution was placed on YPX, YPD75 and YP-hydrolysate plates. The plates were incubated at 30°C for at least two days and evaluated by visual inspection.

### Analysis of metabolites and cell dry weight

Samples for extracellular metabolites were analyzed by high performance liquid chromatography using Aminex HPX-87H column with 30 x 4.6 mm Cation-H Biorad micro-guard column maintained at 45°C. 5 mM H_2_SO_4_ was used as an eluent at a flow rate of 0.6 ml min^-1^. Glucose, xylose, xylitol, acetic acid, glycerol, and ethanol were detected using RI detector maintained at 35°C and HMF, furfural, vanillin and syringaldehyde were detected using UV detector at 210 nm. The sugars in spruce hydrolysate and samples from chemostat cultures were analyzed by high performance anion exchange chromatography using 4 x 250 mm Dionex CarboPac PA1 column with 4 x50 mm guard column maintained at 30°C. Eluent A: 300 mM NaOH, eluent B: 100 mM NaOH+85 mM sodium acetate were used for elution at a flow rate of 1 ml min^-1^. Monosaccharides including arabinose, galactose, glucose, xylose and mannose were detected using pulsed amperometric detector. Optical density (OD) and cell dry weight were used as an estimate of cell concentration. OD was measured at 650 nm using the cell free medium at the point of sampling as background. Cell dry weight was determined in duplicates by filtering 5–10 ml of culture through a pre-weighed 0.45μm hydrophilic polyethersulfonate filter (Sartorius Stedim Biotech, Germany). After removal of medium the filters were washed with milliQ water and dried in microwave at 125W for 15 min. The filters were kept in a desiccator over night before they were weighed again. All yields and rates were calculated in the exponential phase identified by natural logarithm of cell dry weight or OD_650_ and culture time. The lag phase time was calculated from the point of intersection of lag phase and exponential phase in a plot of natural logarithm of OD_650_ and culture time.

### Enzyme activities

XR and XDH assays were perfomed in SARSTEDT 96-well microtiter plates with a FLUOstar Omega multi-mode microplate reader (BMG LABTECH, Ortenberg, Germany). Culture aliquots stored at −80°C were used to inoculate minimal medium containing 20 g l^-1^ glucose and xylose, respectively in shake flasks. The concentration of salts, trace elements and vitamins were exactly the same as used in the medium for evolutionary engineering in shake flasks. Cells were harvested in xylose phase (10 h after glucose was consumed), washed twice with water and resuspended in disintegration buffer, 0.1 M triethanolamine buffer (pH 7.0), containing 1 mM phenylmethylsulfonyl fluoride, 0.5 mM dithiothreitol, and 0.5 mM EDTA and glass beads. The suspension was vortexed in FastPrep-24 instrument (MP Biomedicals, France) for 20 s and kept in ice for 2 min and this cycle was repeated for six times. Later, the suspension was centrifuged at 20,000 g, 5 min, 4°C and the supernatant was used for enzymatic assays. The total protein concentration was determined using Bio-Rad protein assay dye reagent (Bio-Rad laboratories, US) with bovine serum albumin as standard. XR and XDH activity were measured as previously described in [23]. Experiments were performed in duplicates of three different concentrations of cell extract. Specific activities are expressed as units per milligram of protein. Units are defined as micromoles of NADPH oxidized or NAD^+^ reduced per minute.

### Nomenclature of evolved cells

Cells evolved in shake flasks were named accordingly as follows, RK_% inhibitor cocktail_round of cultivation, for instance, strain RK40-3 was evolved in 40% inhibitor cocktail for three rounds of cultivation. For the cells that were exposed to UV were named accordingly as follows, RKU_seconds of UV exposure_cycle of cultivation in 60% inhibitor cocktail, for instance, RKU90-3 was exposed to UV for 90 seconds and evolved in 60% inhibitor cocktail for three rounds of cultivation. Cells evolved in chemostat were named as follows, KE1_no. of. isolates, for instance, KE1-5 was the 5^th^ isolate of evolution in the chemostat culture.

## Abbreviations

HMF, 5-hydroxymethyl-2-furaldehyde; XR, Xylose reductase; XDH, Xylitol dehydrogenase; XYL1 and XYL2, Genes encoding for xylose reductase and xylitol dehydrogenase, respectively; ADH, Aldehyde dehydrogenase; PGK, Phosphoglycerate kinase; HPLC, High performance liquid chromatography; HPAEC, High performance anion exchange chromatography; UV, Ultraviolet; YPD, Yeast peptone dextrose; YPD75, YP-hydrolysate: agar plates with inhibitors and spruce hydrolysate, respectively.

## Competing interests

EA was employed by Taurus energy AB during the work. LO does consultancy for Taurus energy AB.

## Authors’ contribution

RK, EA and LO participated in the design of the study. RK and EA performed the experimental work and wrote the manuscript. LO and EA participated in the conception of the study, commented on the manuscript. All the authors read and approved the final manuscript.
